# Ex vivo hepatic venography for hepatocellular carcinoma in livers explanted for liver transplantation

**DOI:** 10.1186/1477-7819-9-111

**Published:** 2011-09-27

**Authors:** Kensuke Miyazaki, Akihiko Soyama, Masaaki Hidaka, Koji Hamasaki, Kosho Yamanouchi, Mitsuhisa Takatsuki, Takashi Kanematsu, Susumu Eguchi

**Affiliations:** 1 Department of Surgery, Nagasaki University Graduate School of Biomedical Sciences, Nagasaki, Japan

## Abstract

**Background:**

Hepatocellular carcinoma (HCC) is supposed to have a venous drainage system to a portal vein, which makes intrahepatic metastasis possible. However, the mechanism of extrahepatic recurrence, including the possibility of a direct route to the systemic circulation from the HCC nodules, remains unclear. Therefore, we performed retrograde hepatic venography for HCC in livers that had been explanted for liver transplantation in order to explore the possible direct connection between the hepatic vein and HCC nodules.

**Methods:**

Of 105 living-donor liver transplantations (LDLT) performed up to July, 2009 at the Department of Surgery, Nagasaki University Hospital, dynamic hepatic venography was performed with contrast media under fluoroscopy for the most recent 13 cases with HCC. The presence of a tumor stain for each HCC case was evaluated and compared with the histological findings of HCC.

**Results:**

Hepatic venography revealed a tumor stain in 2 of 13 cases (15%). Neither showed any microscopic tumor invasion of HCC into the hepatic vein. In the other 11 cases, there were 4 microscopic portal venous invasions and 2 microscopic hepatic venous invasions. No patients have shown HCC recurrence in follow-up (median period, 13 months).

**Conclusion:**

Using *ex vivo *hepatic venography, a direct connection to the hepatic vein from HCC in whole liver was revealed in 2 cases without demonstrated histopathological invasion to hepatic vein for the first time in the literature. The finding suggests that there is direct spillage of HCC cells into the systemic circulation via hepatic vein.

## Introduction

Hepatocellular carcinoma (HCC) is one of the most common malignant tumors and the third most common cause of cancer-related death in the world [1]. Despite recent advances in treatments of HCC, the long-term survival of patients with HCC is still unsatisfactory [2]. Intrahepatic or extrahepatic recurrence usually develops, even after a curative liver resection or a total hepatectomy for orthotopic liver transplantation. There are two mechanisms that are well known for intrahepatic recurrence of HCC: multicentric carcinogenesis due to the underlying liver disease and intrahepatic metastasis with venous drainage to a portal vein [3]. On the other hand, the mechanism of extrahepatic recurrence of HCC is still controversial, with the possibility of a direct route from HCC nodules to the systemic circulation still unconfirmed. According to previous reports, the predictors for extrahepatic recurrence of HCC are the size and number of tumors, vascular invasion, and elevated tumor markers [4-8]. However, even cases with small and single HCC lesions sometimes develop extrahepatic recurrence.

In this study, we performed hepatic venography for HCC in explanted livers for liver transplantation, in order to explore the direct connection between the hepatic vein and HCC nodules.

## Methods

### Patients

One hundred five living-donor liver transplantations (LDLT) were performed up to July, 2009 at the Department of Surgery, Nagasaki University Hospital. Of these 105 LDLTs, we performed hepatic venography on explanted livers of the most recent 13 cases, which detected HCC lesions preoperatively and/or postoperatively. There were 9 males and 4 females with a median age at LDLT of 59 years (range; 52-68 years) (Additional file 1, Table S1).

### Ex vivo hepatic venography

**Figure 1:**
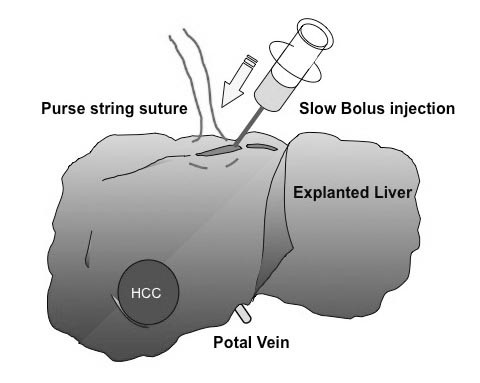
**Methods of hepatic venography**. ***1 ***Purse-string suture around the orifice of the hepatic vein (to prevent back flow) ***2 ***Cannulation into the hepatic vein ***3 ***Slow bolus injection of the contrast media ***4 ***X-ray imaging under fluoroscopy.

Using livers explanted for LDLT, hepatic venography was performed with contrast media (Urografin, Nihon Schering, Osaka, Japan) under fluoroscopy. First, a purse-string suture was placed around the orifice of the right hepatic vein and the hepatic venous trunk of the middle and left hepatic vein, respectively, to prevent the backflow of the contrast media. Second, a plastic needle was cannulated into the hepatic vein and tightened up with the purse-string suture. Third, the contrast media was injected into the hepatic vein by a slow retrograde bolus injection with very low pressure manually (Figure 1). Thereafter, X-ray images were taken from several different angles in a series.

### Evaluation of hepatic venography

We defined the venographic positve case as the presence of a tumor stain corresponding to an HCC nodule contiguous with hepatic vein, and smoothly filled with contrast media without any resistance. The presence of a tumor stain corresponding to an HCC nodule was judged to be "positive", while the absence of a tumor stain was judged to be "negative" on the serial x-ray images. The judgment was made by two or three surgeons.

### Statistical analysis

Fisher's exact test was used for the data analysis. A level of *P *< 0.05 was considered to indicate statistical significance.

## Results

### Ex vivo hepatic venography

**Figure 2:**
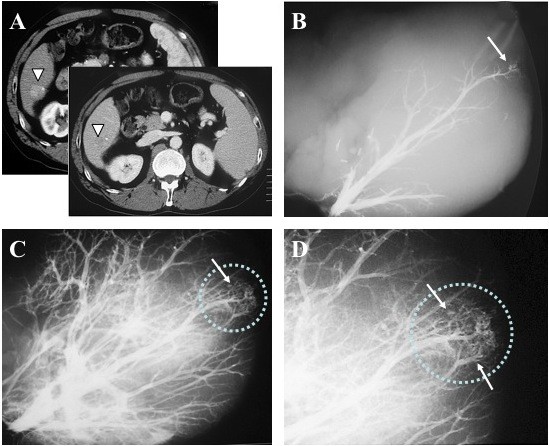
**A hepatic venography-positive case**. A 65-year-old male with a 2.2-cm HCC at segment 6 (white arrowheads on CT). The broken circle indicates the site of the tumor. Hepatic venography shows tumor stains (arrows) corresponding with an HCC nodule. **A**: Enhanced CT **B**: Early phase image of hepatic venography **C**: Late phase image of hepatic venography **D**: magnified image of the late phase.

**Figure 3:**
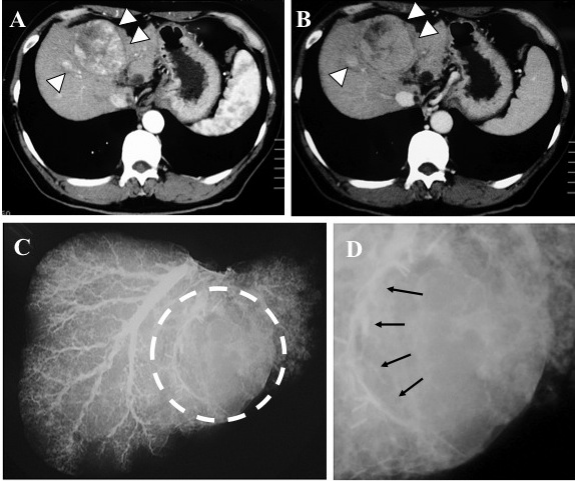
**A hepatic venography-negative case**. A 58-year-old male with multiple HCCs. A 6.2-cm HCC at segment 4 (white arrowheads). The broken circle indicates the site of the tumor. Hepatic venography shows no tumor stain but venous compression from the tumor (arrows). **A**: Enhanced CT, early phase **B**: Enhanced CT, delayed phase **C**: Image of hepatic venography **D**: Magnified image of hepatic venography.

In 2 of 13 cases (15%), tumor stains were confirmed in a corresponding lesion to indicate the location of an HCC nodule. One positive case was a 65-year-old male with 2.2-cm HCC located at segment six, associated with hepatitis C virus-related cirrhosis. The tumor stain from the right hepatic vein was clearly detected with contrast media by retrograde hepatic venography (Figure 2). The other positive case was a 68-year-old male having a 3.2-cm HCC at segment six. A tumor stain was also seen by retrograde hepatic venography from the right hepatic vein (image not shown). The other 11 cases were judged negative by hepatic venography (Additional file 2, Table S2). Though negative cases showed no tumor stains with contrast media, they did show venous compression from the tumor (Figure 3).

### The relationship between hepatic venography and histopathological findings

Histopathologically, there were 4 portal venous invasions and 2 hepatic venous invasions among the 13 cases. Of the 2 positive hepatic venography cases, one showed portal venous invasion, but neither showed microscopic hepatic venous invasion. No statistically significant relationship was found between tumor stains by ex vivo hepatic venography and microscopic hepatic venous invasion in positive hepatic venography cases.

### Outcomes after LDLT

Five of the patients who underwent hepatic venography died. Two patients died of sepsis, and the other three died due to liver failure. However, no recurrent HCC was found in the follow-up (median period, 25 months) of the 13 cases, including the two patients with venographically demonstrable HCC, who had follow-up periods of 35 and 25 months after LT, respectively, at this writing (Additional file 2, Table S2).

## Discussion

In this study, we have demonstrated a direct connection between primary HCC nodules and the systemic circulation (vena cava) by retrograde hepatic venography with livers explanted for liver transplantation. To our knowledge, this is the first report to visualize the direct communication from HCC nodules to the systemic circulation in explanted whole livers from liver transplantation. In this study, 2 of 13 cases showed tumor stains, which indicate direct venous drainage to the hepatic vein (or vena cava), by hepatic venography. The stains might represent HCC cells or tumor thrombi spilled from the primary HCC into systemic circulation and thereby likely to be carried to distant organs by the bloodstream. In spite of the presence of a direct connection to the hepatic veins, neither of the two positive cases showed microscopic hepatic venous invasion. These results suggest that the route of the cancer cells into the vessels could be independent of histopathological invasion.

Based on previous reports, various factors are thought to contribute to extrahepatic recurrence; for instance, Funaki et al. reported hematogenous spreading of HCC cells from the primary tumor [9]. Recently, some studies have reported that adhesion molecules, such as E-cadherin [10,11] or CD44 [12,13], play an important role in the extrahepatic recurrence of HCC after hepatectomy or liver transplantation. Other reports have indicated that the presence of cancer stem cells is a key factor [14,15]. In any case, cancer cells from the primary lesion likely migrate into the bloodstream of systemic circulation to form metastatic foci in distant organs.

Moreover, several factors seem to be involved in the occurrence of distant metastasis, such as *1) *escape from local immunity, *2) *connection to systemic circulation, *3) *spilling of HCC cells from the primary lesion into the bloodstream, *4) *escape from the host immune surveillance systems, *5) *adhesion to another organ, and *6) *growth. A recent study represents a case of metastasis without pathological venous invasion. Sugino et al. [16,17] described sinusoidal angiogenesis as a non-invasive mechanism of blood-borne metastasis in HCC; i.e., an invasion-independent metastasis pathway. This may suggest that patients after liver transplantation need particularly vigilant observation for extrahepatic recurrence because of their immunosuppressive states.

In addition, the possibility of intrahepatic metastasis as well as extrahepatic metastasis via systemic circulation has also been reported [18]. Thus, patients having HCC with a direct connection to systemic circulation should be monitored, not only for extrahepatic recurrence but also for intrahepatic recurrence, even after liver transplantation.

There are some reports of efferent vessels of HCC. Mitsunobu et al. [19] demonstrated that the portal vein serves as an efferent vessel in advanced HCC by direct injection of radiopaque media into HCC nodules of resected specimens. Other reports have made similar conclusions from different points of view, namely, histopathological study [20] or color Doppler imaging examination using ultrasonography [21]. Those previous reports suggested the following mechanism. The efferent vessel of hepatic tumors is basically the hepatic vein; blood from the HCC still flows out to the hepatic vein at its early stage. With the progress of HCC, the portal vein also acts as an efferent vessel. It is supposed that a capsule is formed as the HCC undergoes dedifferentiation, resulting in regurgitation of blood to the portal vein with the rising internal pressure of HCC nodule. This causes intrahepatic metastasis through the portal vein as well.

In regard to the outcomes of LDLTs for the 13 patients, there has been no recurrence of HCC so far. This may be due to the fact that the follow-up periods are not very long (37 months at most), and that all cases except one were within the Milan criteria [22].

## Conclusion

Hepatic venography of 2 of 13 livers explanted for HCC-related LDLT revealed a direct connection between primary HCC nodules and the hepatic vein. Such cases should be strictly observed for extrahepatic and intrahepatic recurrence, even in cases within the Milan criteria and without microscopic hepatic venous invasion.

## Competing interests

The authors declare that they have no competing interests.

## Authors' contributions

SE designed and coordinated the study. KM, AS, MH, KH performed and carried out the hepatic venography, and KM wrote the manuscript. All authors evaluated the results of the hepatic venography. KY, MT, TK, SE supervised in critically reviewed the manuscript. All authors contributed significantly to this work, and approved the final manuscript.

## Supplementary Material

Additional file 1 **Table S1**. Patients Characteristics. Click here for file

Additional file 2 **Table S2**. Tumor characteristics and clinical outcomes of patients who underwent hepatic venography. Click here for file
